# Elastofibroma of the Face: A Case Report

**Published:** 2015-03

**Authors:** Sorena Fardisi, Mohammad Javad Ashraf, Mohammad Reza Zarei, Negar Azarpira, Maryam Raoof, Sara Amanpour

**Affiliations:** 1 Postgraduate Student, Dept. of Oral and Maxillofacial Surgery, School of Dentistry, Shiraz University of Medical Sciences, Shiraz, Iran;; 2 Dept. of Pathology, School of Medicine, Shiraz University of Medical *Sciences*, Shiraz, Iran;; 3 Dept. of Oral Medicine, School of Dentistry, Kerman University of Medical Sciences, Kerman, Iran;; 4 Organ Transplant Research Center, Shiraz University of Medical Sciences, Shiraz, Iran;; 5 Laboratory of Molecular Neuroscience, Neuroscience Research Center, Institute of Neuropharmacology, Kerman University of Medical Sciences, Kerman, Iran, Dept. of Biology, Faculty of Sciences, Shahid Bahonar University, Kerman, Iran, Dept. of Endodontics, School of Dentistry, Kerman University of Medical Sciences, Kerman, Iran;; 6 Dept. of Oral and Maxillofacial Pathology, School of Dentistry, Kerman University of Medical Sciences, Kerman, Iran, Oral and Dental Disease Research Center, Kerman University of Medical Sciences, Kerman, Iran;

**Keywords:** Elastofibroma, Face, Case Report

## Abstract

Elastofibroma is a rare neoplasm that characteristically occurs in subscapular area in response to microtrauma. There are some reports of this tumor in other sites of the body but, up till now, there has been no report of elastofibroma in the face. A 20-year-old man presented with a slow growing painless mass in the face without any history of trauma. Histopathologic examination revealed a soft tissue mass composed of eosinophilic fibers admixed with aggregation of fat cells, capillary blood vessels, and fibroblasts. Elastic stain and Masson’s trichrome stain confirmed the nature of elastic and collagen fibers. It was a case of elastofibroma in the face.

## Introduction

Elastofibroma is a rare benign connective tissue neoplasm that mostly occurs in subscapular area of elderly patients, deep in the serratus anterior muscle.[1] There are reports of this tumor arising in other sites such as hand,[2] foot,[3] thigh,[4] gastrointestinal tract,[5-6] neck,[7]and mouth.[8-10]The pathogenesis of elastofibroma is still unknown but it may result from a reactive response to repetitive microtrauma.[7] To the best of our knowledge, elastofibroma has not been reported in the face so far. In this paper, we describe a case of elastofibroma in the face of a 20-year-old man.

## Case report

**Figure 1:**
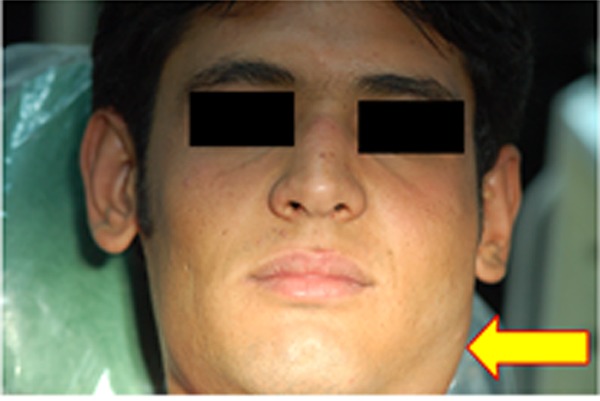
Clinical view; a non-tender, firm, and movable mass in left parotid area

A 20-year-old man was referred for a painless mass in the left parotid area that has been growing slowly for about 3 years. Clinical examination revealed a firm non-tender mass of approximately 2×3 cm^2^ which was easily movable (Figure 1).

**Figure 2:**
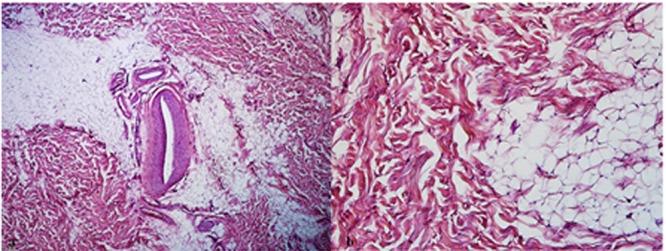
a: Microscopic picture of elastofibroma, mainly composed of fibers admixed with aggregation of fat cells and capillary blood vessels (Hematoxylin-Eosin, original magnification ×100)     b: Elastofibroma, (Hematoxylin-Eosin, original magnification ×400)

The overlying skin was normal. There was no specific history of trauma to the area of the lesion and the patient did not report a family history of similar lesions. The differential diagnosis included a preauricular or sebaceous cyst and enlarged lymph nodes. Fine needle aspiration of the lesion revealed few benign looking spindle shape cells in favor of a benign fibrous lesion. The mass was completely excised under local anesthesia and histopathologic examination revealed a soft tissue tumor mainly composed of irregular crinkled eosinophilic fibers with corrugated margins and variable shapes and sizes. Aggregation of fat cells, capillary-sized blood vessels and fibroblasts were also observed within the specimen (Figures 2).

**Figure 3:**
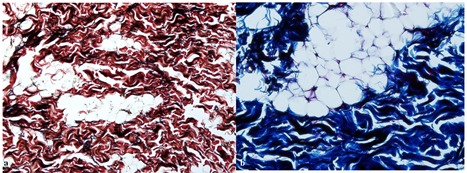
a: Elastin stain shows high density of elastic fibers (Verhoeff-Van Gieson stain, original magnification ×400)  b: Masson-trichrome stain identified collagen fibers within the lesion (Masson-trichrome stain, original magnification ×400)

A Verhoeff-Van Gieson stain and Masson’s-trichrome stain identified elastic fibers and abundant collagen fibers, respectively (Figures 3). Based on microscopic findings and special staining, the diagnosis of elastofibroma was made. Up to now, there has been no clinical sign of recurrence during the 3-year follow-up of the patient.

## Discussion

Elastofibroma is a benign process involving the subscapular region in most cases. However, isolated lesions have been reported in thigh, lip, deltoid muscle, and stomach.[1] It was first described by Jarvi and Saxen in 1961.[11] The lesion is typically seen in females over the age of 50.[12-13] Frictional trauma has been suggested as the etiopathogenesis of this lesion.[8] A neoplastic etiology is also possible in some cases based on unusual clinical presentations and molecular studies.[14] Surgical removal is the treatment of choice for this lesion and there has been no report of malignant transformation.[9]

Microscopically, collagen bundles alternate with numerous degenerative fibers in irregular shapes are seen which stain strongly with elastic stain that is allied to mucoid materials, fibroblasts, and small collections of mature lipocytes.[15-16] In the case of elastofibroma reported by Manchandu et al., the elastic fibers showed flower-like appearance with serrated borders.[14] Elastic fibers in this tumor seem to be the result of altered elastogenesis caused by frictional trauma.[8] However, several cases of elastofibroma have been reported in different locations without having trauma.[17-2]1 It may be better explained by a genetic predisposition.[8] The relationship between elastofibroma and genetic factors was first described by Nagamine et al.[5] They reported the influence of hereditary factors on one third of 170 patients.[5]

To the best of our knowledge, this is the first case of elastofibroma that has been presented as a facial mass. Thus, this benign soft tissue tumor should be considered in the differential diagnosis of painless soft tissue masses in the face. 
